# *In Vitro* and *In Vivo* Cytogenotoxic Effects of Hot Aqueous Extract of *Achyrocline satureioides* (Lam.) DC.

**DOI:** 10.1155/2015/270973

**Published:** 2015-05-11

**Authors:** L. N. Cariddi, M. C. Sabini, F. M. Escobar, R. Bacchetti, I. Montironi, C. Merckis, E. B. Reinoso, S. Núñez Montoya, S. M. Zanon, L. R. Comini, L. I. Sabini

**Affiliations:** ^1^Departamento de Microbiología e Inmunología, Universidad Nacional de Río Cuarto, Ruta 36, Km 601, Río Cuarto, C5800 Córdoba, Argentina; ^2^Consejo Nacional de Investigaciones Científicas y Tecnológicas (CONICET), Rivadavia 1917, C1033AAJ Buenos Aires, Argentina; ^3^Área de Microscopía Electrónica, Universidad Nacional de Río Cuarto, Ruta 36, Km 601, Río Cuarto, C5800 Córdoba, Argentina; ^4^Farmacognosia, Departamento de Farmacia, Universidad Nacional de Córdoba (IMBIV-CONICET), Ciudad Universitaria, C5000 Córdoba, Argentina

## Abstract

In this work we extend the toxicological studies of hot aqueous extract of* A. satureioides* (As-HAE) evaluating cytotoxic and apoptotic effects on human peripheral blood mononuclear cells (PBMCs). We also determine genotoxic action of this extract* in vivo*. In addition, the extract was chemically characterized. Finally, we established a comparison with previous data of cold aqueous extract. The As-HAE induced cytotoxicity on PBMCs determined by trypan blue dye exclusion (CC_50_ = 653 *μ*g/mL) and MTT (CC_50_ = 588 *μ*g/mL) assays being more toxic than cold extract. However, As-HAE as well as cold extract did not induce apoptosis measured by Hoechst 33258 staining, TUNEL assay, and DNA fragmentation analysis. The* in vivo* micronucleus test showed that As-HAE exerted cytogenotoxic effects on bone marrow of mice, contrary to what was observed with cold extract. The chemical study of As-HAE allowed identifying the flavonoids found in cold extract: luteolin, quercetin, and 3-*O*-methylquercetin, but at higher concentrations. We suggest that toxic effects induced by As-HAE could be due to high concentrations of these flavonoids. Given that As-HAE is the most used in folkloric medicine, its administration should be controlled in order to prevent potential cell damage.

## 1. Introduction

*Achyrocline satureioides* (Lam.) DC. is an important medicinal species which belongs to Asteraceae family. This plant, commonly known as “Marcela,” is native from America and it extends throughout the continent as well as in Europe and Africa.* A. satureioides* is used extensively in popular medicine as infusion, decoction, or maceration. In addition, this vegetal species is used in the manufacture of bitter beverages consumed as aperitifs and in numerous medicinal products, which are currently marketed in South America, United States, and Europe [1]. The phytochemical investigation of this plant species has shown that the flavonoids quercetin, luteolin, and 3-*O*-methyl quercetin are the main constituents of its polar extracts. In addition, a relationship between these flavonoids and the pharmacological properties attributed to this plant was found [2, 3]. Some activities related to these flavonoids are antioxidant, anti-inflammatory, antihyperglycemic, and antitumor for luteolin [4, 5]; antioxidant and anti-inflammatory for quercetin [6, 7]; and antiviral activity reported for 3-*O*-methylquercetin [8]. All these researches show the great ethnobotanical potential of* A. satureioides* and reveal the interest of quantifying these constituents in phytopharmaceutical preparations, as well as food preparation. Furthermore, although the flavonoids are often described as nontoxic compounds, some studies have demonstrated that they are capable of influencing a variety of cellular functions by modulating cell signaling and proliferation [9] and under certain conditions may exhibit toxic activity by production of free radical species [10].

For this reason, is necessary to study the toxicity of extracts obtained from* A. satureioides,* mainly on normal cells, in order to evaluate the potential risk for human. In previous studies, we have demonstrated that cold aqueous extract of* A. satureioides* was not able to induce cytogenotoxic effects at concentrations used popularly [11]. Regarding the hot aqueous extract, the most used in folkloric medicine, a previous study showed that it was toxic on Vero cells [12].

Therefore, the present study aimed to extend the toxicological studies of hot aqueous extract from* A. satureioides* by evaluating the cytotoxic and apoptotic effects on another cell line: human peripheral blood mononuclear cells (PBMCs), as well as by determining the genotoxic action of this extract by two* in vivo* methods:* Allium cepa* L. test and micronucleus test on bone marrow of mice. In addition, the extract was chemically characterized. Finally, we established a comparison with results obtained in previous studies with cold aqueous extract.

## 2. Materials and Methods

### 2.1. Vegetal Material

Aerial parts of* Achyrocline satureioides* (Lam.) DC. (Asteraceae) were collected from Alpa Corral, southern Córdoba hills (32°41′S; 64°43′W; 800 m sea level), Argentina, in May 2007. This plant was identified and taxonomically classified by Dr. Luis Del Vitto in the Facultad de Farmacia y Bioquímica of the Universidad Nacional de San Luis, and a voucher specimen was stored in the UNSL herbarium as file #6362.

#### 2.1.1. Preparation of Plant Extract

Twenty grams of dried plant material was extracted with 1 L of doubly distilled water at 70°C for 48 h. The final product, hot aqueous extract of* A. satureioides* (As-HAE), was lyophilized (Lyophilizer Labconco freeze dry system 4.5, Labconco Corporation, Kansas City, USA) and stored at −20°C. Before using, it was dissolved in phosphate buffered saline (PBS) to obtain an initial concentration of 2.5 mg/mL of extract.

### 2.2. Isolation of Human PBMCs

Peripheral blood was drawn from healthy volunteers (18 to 25 years old). PBMCs were isolated from blood samples using Histopaque-1077 centrifugation (Sigma Aldrich, St. Louis, USA). Cell viability was determined by trypan blue dye exclusion assay using an optimal suspension of 1 × 10^6^ cells/mL [13]. The study was approved by Comité de Ética de la Investigación Científica (COEDI), Universidad Nacional de Río Cuarto.

#### 2.2.1. Cell Viability Assay

PBMCs (2 × 10^5^/well) in a final volume of 200 *μ*L were cultured in sterile 96-well microplate containing RPMI-1640, supplemented with 25 mM Hepes, 2 mM L-glutamine, 5% FCS, 50 mM 2-ME, 100 *μ*g/mL streptomycin, 100 *μ*g/mL penicillin, and 100 *μ*g/mL neomycin. Cells were exposed to different concentrations of As-HAE (0, 10, 50, 100, 200, 400, 600, 800, and 1000 *μ*g/mL). Cell cultures with only RPMI-1640 were used as control. The system was incubated at 37°C with 5% CO_2_ and humidity for 24 h. After that period, cell viability was evaluated by two independent methods: trypan blue dye exclusion using Neubauer chamber [14] and colorimetric MTT assay [15]. Four replicate wells for each exposure in six independent plates were performed.

*(1) Apoptosis Analysis by Hoechst Staining.* Cell morphology was evaluated by fluorescence microscopy followed by Hoechst 33258 DNA staining (Sigma Aldrich, St. Louis, USA), as described by Montaner et al. [16] with modifications. Briefly, PBMCs were cultured as previously described and exposed to As-HAE. In addition, cells cultured with medium only and cells treated with hydrogen peroxide (1 mmol/L) were used as negative and positive controls, respectively. After that, cells were centrifuged and fixed with cold methanol (−20°C). Then, cells were stained with Hoechst 33258 (20 *μ*g/mL final concentration) and incubated for 5 min at room temperature in darkness. Cells were examined with light microscope (Axiophot, Carl Zeiss, Germany) attached to the image-analysis system (Powershot G6, 7.1 megapixels, Canon INC, Japan, with software AxioVision Release 4.6.3, Carl Zeiss, Germany). Apoptotic cells were identified by observing characteristic features of apoptosis (e.g., nuclear condensation, formation of membrane blebs, and apoptotic bodies). 

*(2) TUNEL Assay*. The number of apoptotic human PBMCs was assessed by TUNEL staining using ApopTag Plus Peroxidase In Situ Apoptosis Kit (Chemicon International, USA), as described by Song et al. [17], with modifications. After each treatment, cells were centrifuged and fixed in slides with acetic acid and MeOH (1 : 4). In addition, cells cultured with medium only and cells treated with hydrogen peroxide (1 mmol/L) were used as negative and positive controls, respectively. Cells were then incubated with 20 *μ*g/mL of proteinase k (Sigma Aldrich, St. Louis, USA) for 15 min at room temperature and treated with 3% hydrogen peroxide for 5 min. The slides were incubated with DNA-terminal deoxynucleotidyl transferase (TdT) at 37°C with humidity for 1 h. After that, the slides were incubated with antidigoxigenin antibody conjugated to peroxidase, which was used to label the incorporated digoxigenin-labeled nucleotides, and added with the substrate supplied by the manufacturer. The slides were counterstained with Harris hematoxylin. Apoptotic cells were then assessed as the percentage of TUNEL-positive cells per 400 cells in each slide using a light microscope (Axiophot, Carl Zeiss, Thornwood, NY) attached to the image-analysis system (Powershot G6, 7.1 megapixels, Canon INC, Japan, with software AxioVision Release 4.6.3, Carl Zeiss, Germany).

*(3) DNA Fragmentation Analysis*. The isolation of fragmented DNA from cells exposed to different concentrations of As-HAE was carried out according to the procedure of Amirghofran et al. [18] with modifications. In brief, cells (2 × 10^5^ cells/well) were treated with every plant extract concentration and then centrifuged (2600 rpm, 15 min). In addition, cells cultured with medium only and cells treated with hydrogen peroxide (1 mmol/L) were used as negative and positive controls, respectively. The pellet was suspended in 0.5 mL of DNA lysis buffer (2% SDS, 10 mM EDTA, 10 mM Tris-HCl, and pH 8.5). The lysate was immediately incubated with 0.1 mg/mL proteinase k (Sigma Aldrich, St. Louis, USA) and 0.5 mg/mL RNAase A (Boehringer Mannheim, Germany) for 3 h at 37°C. A volume of 200 *μ*L of 3 M NaCl was added and the system was centrifuged at 3000 rpm for 15 min. After isopropanol addition, the DNA was precipitated with 70% ethanol. The samples were loaded into 2% agarose gel and electrophoresis was carried out. The DNA band pattern was visualized under UV light using ethidium bromide staining.

### 2.3. Genotoxicity Assay by* Allium cepa* L. Test

Although* Allium cepa* L. test is a simple* in vivo* model, it has proven to be an efficient prescreening technique for later cytogenotoxic studies. We decided to perform this test as a preliminary test to the micronucleus test in bone marrow of mice.

*Allium test* was developed as described by Fiskesjö [19] with modifications. Qualitative and quantitative changes, macro- and microscopic alterations, induced by treatment with As-HAE in plant cells were assessed. Onion root tips of* Allium cepa* L. grown in mineral water, in darkness, with aeration and constant temperature (25 ± 0.5°C) were employed. As-HAE was assayed at 0.5, 1, 2, 3, and 4 mg/mL. Positive (paracetamol 0.3 mg/mL) and negative (mineral water) controls were included in the system. Extract concentrations were applied for different times: 2 and 5 days. The treatment of 2 days was followed by 3 days with water (reversion). At the end of each treatment, 2 root tips from each bulb were cut and fixed in a mixture of absolute alcohol : glacial acetic acid (3 : 1, v/v). These roots were hydrolyzed in 1 N HCl for 5 min and washed with distilled water. The root tips were then squashed on slides, stained with acetocarmine for 10 min, and cover slips were carefully lowered to exclude air bubbles. The cover slips were sealed on the slides with clear fingernail polish. These roots were reserved to evaluate cytogenetic abnormalities (microscopic features). Eight slides from each concentration and controls (at 1000 cells per slide) were prepared and analyzed at ×1000 magnification for evaluation of chromosomal aberration induction. The mitotic index was calculated as the ratio between the number of cells in division and 1000 cells observed. In addition, indices of phases were also calculated. The percentage frequency of aberrant cells was calculated based on the number of aberrant cells per total cells scored at each extract concentration.

### 2.4. Animals

Male and female Balb/c mice aged 8–12 weeks (weighing 20–25 g) were obtained from the Bioterio Central of the Universidad Nacional de Río Cuarto. Animals were maintained in a temperature and humidity controlled room, with 12 h light-dark cycles, and were allowed food and water* ad libitum*. All experimental procedures were conducted in accordance with recent legislation. This study was approved by the Comité de Ética de la Investigación Científica (COEDI), Universidad Nacional de Río Cuarto.

### 2.5. Genotoxicity Assay by Micronucleus Test

This trial was carried out using the micronucleus test in mouse bone marrow as described by Schmid [20], with modifications. Briefly, Balb/c mice were separated into groups of 6 animals (3 males and 3 females) and injected intraperitoneally following independent trials. Three concentrations of As-HAE diluted in saline solution (100, 200, and 500 mg/kg) were used. These concentrations were chosen based on a previous study with cold aqueous extract of* A. satureioides* [11]. The negative control group received saline solution by the same route, and the positive control group received 30 mg/kg body weight of cyclophosphamide (Sigma Aldrich, St. Louis, USA). The animals were sacrificed by cervical dislocation at 24 h after injection. Bone marrow samples from femoral bone obtained with FCS were fixed with ethanol and stained with May-Grünwald and Giemsa. In order to evaluate the genotoxic properties induced by the plant extract, the presence of erythrocytes with micronuclei (MN) was observed in a total of 2000 polychromatic erythrocytes (PCE) per animal. Furthermore, to obtain a toxicity grade of As-HAE on bone marrow, the toxicity index (TI) was calculated by the PCE/NCE ratio in 1000 cells.

### 2.6. Identification and Quantification of Flavonoid Derivatives by HPLC-ESI-MS/MS

#### 2.6.1. Preparation of a Flavonoids Enriched Fraction from the As-HAE

The lyophilized As-HAE (32 mg) was dissolved in H_2_O (3 mL) and was exhaustively extracted with diethyl ether (Ether, three times with 2 mL). Thus, a rich-extract in the flavonoids present in the As-HAE complex matrix was obtained. The resulting ethereal phase (As-HAE-Ether) was evaporated to dryness.

#### 2.6.2. Sample Preparation

Three individual solutions of the As-HAE-Ether in MeOH (1.7 mg/mL) were prepared to carry out its qualitative and quantitative analysis by HPLC-ESI-MS/MS. MeOH-HPLC grade (Merck) was used in all samples, which were filtered through a Millipore membrane (0.45 *μ*m) before HPLC analysis.

#### 2.6.3. HPLC-ESI-MS/MS Instruments and Chromatographic Conditions

An Agilent Series 1200 LC System (Agilent, USA) attached to a MicrOTOF Q II (Bruker Daltonics, USA) was used for HPLC-ESI-MS/MS analysis. The HPLC system consisted of a micro vacuum degasser, binary pumps, an autosampler (40 *μ*L sample loop), a thermostated column compartment, and a UV-Visible diode array detector. The mass spectrometer detector is equipped with electrospray ion source and qTOF analyzer. It was used in MS and MS/MS mode for the structural analysis of phenolic compounds and flavonoids. HPLC analysis was performed on a thermostated (40°C) Hypersil 5 column C_18_ (30 × 4.6 mm, Phenomenex) at a 0.4 mL/min flow rate using MeOH-formic acid 0.16 M (53 : 47) as mobile phase [11]. The injection volume was 40 *μ*L.

The UV-Visible detection was performed at 362 nm for flavonoids. MS detection was used for quantification by means of the external calibration method [11]. Calibration curves of the standard compounds were prepared by appropriate dilutions with MeOH from the stock solutions: luteolin (1.9 × 10^−4^) (Merck) and quercetin (5.6 × 10^−4^) (Merck), and they were filtered on Millipore membrane before use. When reference compounds were not available, like in the case of 3-*O*-methylquercetin, the calibration curve of a structurally related substance (quercetin) was used [11]. Compounds concentrations were calculated in triplicate and reported as means ± standard deviation in each case.

### 2.7. Statistical Analysis

All the values obtained in the assays were expressed as averages with standard deviations. The data obtained from toxicity assays were evaluated using GraphPad Prism version 5.00.288 (San Diego, USA, 2007) and compared with one-way analysis of variance (ANOVA) and the Tukey multiple comparison test. The differences were considered to be statistically significant at *P* < 0.05.

## 3. Results

**Figure 1:**
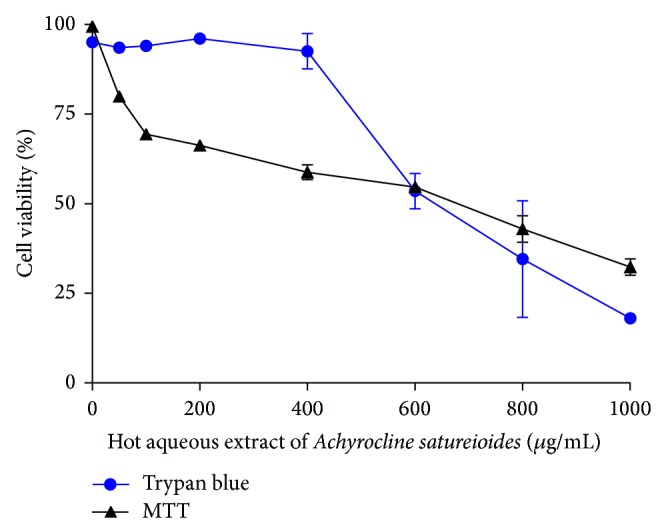
Viability of human PBMCs from healthy individuals exposed to different concentrations of hot aqueous extract of* Achyrocline satureioides *(As-HAE) for 24 h. Cell viability was evaluated by trypan blue dye exclusion and MTT methods. Each value represents the mean ± SD.

The effect of As-HAE on viability of human PBMCs from healthy individuals was studied. A dose-dependent decrease in the number of viable cells was observed when both trypan blue dye exclusion method and MTT assay were conducted, and the cytotoxic concentrations 50% (CC_50_) were 653 and 588 *μ*g/mL, respectively (Figure 1).

**Figure 2:**
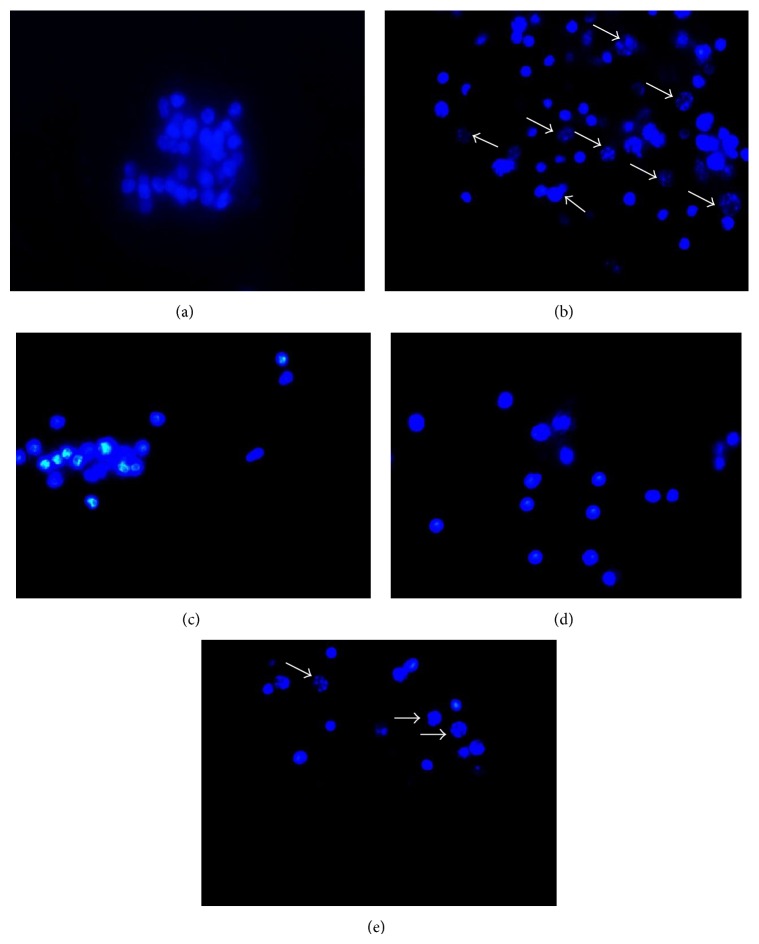
Microphotographs of the nuclear morphology of normal human PBMCs treated with hot aqueous extract of* Achyrocline satureioides *(As-HAE) and stained with Hoechst 33258 (100x). (a) Medium alone (control), (b) cells treated with hydrogen peroxide (1 mmol/L), (c) As-HAE (10 *μ*g/mL), (d) As-HAE (600 *μ*g/mL), and (e) As-HAE (1000 *μ*g/mL). Arrows show apoptotic cells. These cells were identified by characteristic features of apoptosis (e.g., nuclear condensation, membrane blebs formation, and apoptotic bodies).

**Figure 3:**
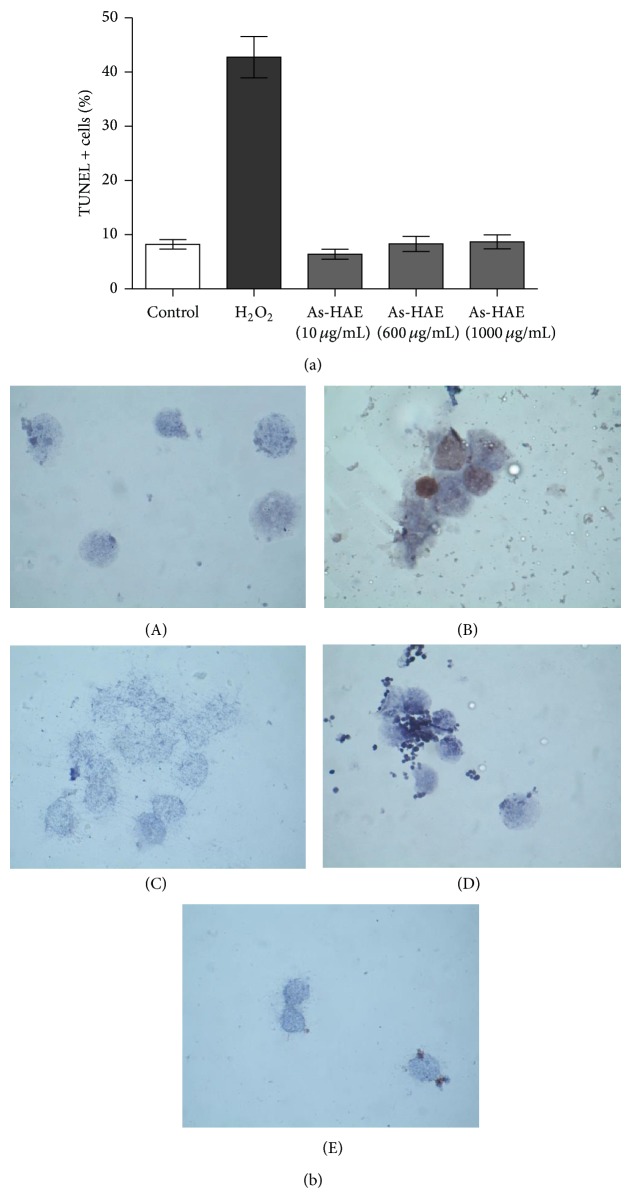
(a) Quantification of apoptotic human PBMCs by TUNEL assay after treatment with hot aqueous extract of* Achyrocline satureioides* (10, 600, and 1000 *μ*g/mL). (b) Photomicrographs of human PBMCs treated with hot aqueous extract of* Achyrocline satureioides* (As-HAE) and stained with TUNEL (100x) using the commercial kit ApopTag Plus Peroxidase In Situ Apoptosis (Chemicon International, USA). (A) Medium alone (control), (B) cells treated with hydrogen peroxide (1 mmol/L), (C) As-HAE (10 *μ*g/mL), (D) As-HAE (600 *μ*g/mL), and (E) As-HAE (1000 *μ*g/mL). Apoptotic cells are shown in brown.

In order to determine whether the cytotoxic effect of As-HAE was due to apoptosis, DNA fragmentation induced by the extract was analyzed. PBMCs morphology was evaluated followed by Hoechst 33258 DNA staining. The nuclei of cells cultured in medium alone were uniformly blue (Figure 2). Fluorescence microscope showed that cells treated with all As-HAE concentrations showed to be similar to the control. However, only some nuclei of the PBMCs treated with the highest concentration of As-HAE (1000 *μ*g/mL) contained small bright blue dots representing chromatin condensation and/or nuclear fragmentation. Other few apoptotic figures were observed such as the formation of membrane blebs and apoptotic bodies (Figure 2). We corroborated these results by TUNEL staining. The percentage of TUNEL-positive cells per 400 cells in cells cultured in medium alone was 7.89 + 0.75% TUNEL + PBMCs. Cells treated with all As-HAE concentrations did not show statistical difference with the negative control (Figures 3(a) and 3(b)). Similarly, any As-HAE concentration assayed showed the typical DNA laddering in agarose gels electrophoresis (data not shown). These results indicate that As-HAE did not cause apoptosis in human PBMCs.

Table 2 summarizes the results of As-HAE on* Allium cepa* roots. The extract concentrations assayed did not alter the number of roots. However, the analysis of roots length showed that every extract concentration causes a significant decrease in length in comparison to the negative control, both at 2 days followed by 3 days with water (reversion) and at 5 days (*P* < 0.05 and *P* < 0.001, resp.).

The mean of roots length at 2 days (with reversion) from all As-HAE concentrations was greater than those obtained at 5 days. Bulbs exposed to As-HAE (0.5 mg/mL) at 2 days (with reversion) showed the highest increase in roots length in comparison to As-HAE (0.5 mg/mL) at 5 days (*P* < 0.001). These results indicate that time of exposure to the extract increased its toxicity and also demonstrate the bulbs ability to recover from damage induced by the extract.

According to macroscopic abnormalities, in both treatments of 2 days (with reversion) and 5 days, there was gelling and necrosis predominance and only the As-HAE concentration of 0.5 mg/mL showed a similar effect to the negative control.

**Figure 4:**
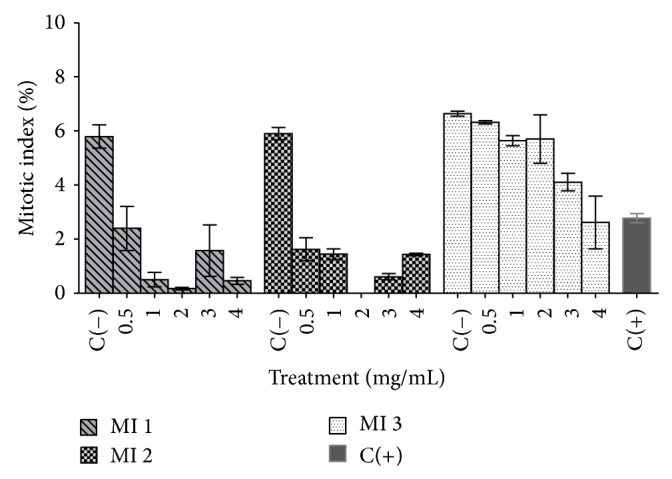
Comparison of mitotic index (MI) of roots of* Allium cepa* L. bulbs treated with hot aqueous extract of* Achyrocline satureioides* (As-HAE). MI 1: mitotic index 1 (treatment with As-HAE for 2 days), MI 2: mitotic index 2 (treatment with As-HAE for 5 days), MI 3: mitotic index 3 (treatment) with As-HAE for 2 days followed by 3 days with water (reversion), C(+): positive control (paracetamol 0.3 mg/mL), and C(−): negative control (mineral water). Each value represents the mean ± SD obtained from four different bulbs (*n* = 4).

Statistical analysis of mitotic index (MI) of bulbs treated with As-HAE revealed that all concentrations assayed at 2 days (MI 1) induced a mitosis decrease with respect to the negative control (*P* < 0.01 for 0.5 mg/mL and *P* < 0.001 for the other concentrations) (Figure 4). The treatments with all As-HAE concentrations at 5 days (MI 2) also induced a mitosis decrease with respect to the negative control (*P* < 0.001) (Figure 4).

None of the As-HAE concentrations for 2 days followed by 3 days with water (reversion) (MI 3) altered mitosis except for the concentration of 4 mg/mL, which induced a mitosis decrease (*P* < 0.05) with respect to the negative control (Figure 4).

The comparative analysis of MI 1 and MI 3 from each concentration showed that after mineral water treatment the MI increased, reaching normal values for 0.5, 1, 2, and 3 mg/mL. These results indicate the roots ability to recover from the toxic effects of As-HAE (Figure 4). On the contrary, the treatment with As-HAE at 4 mg/mL induced irreversible alterations.

**Figure 5:**
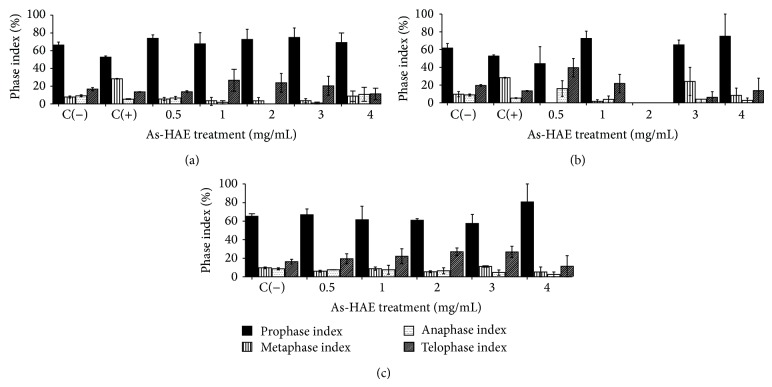
Phase index of cell division in roots treated with hot aqueous extract of* Achyrocline satureioides* (As-HAE). (a) MI 1, (b) MI 2, and (c) MI 3. MI 1: mitotic index 1 (treatment with As-HAE for 2 days), MI 2: mitotic index 2 (treatment with As-HAE for 5 days), MI 3: mitotic index 3 (treatment) with As-HAE for 2 days followed by 3 days with water (reversion), C(+): positive control (paracetamol 0.3 mg/mL), and C(−): negative control (mineral water).

Phase index analysis of bulbs treated with all concentrations of As-HAE at 2 and 5 days and 2 days with reversion did not show statistically significant differences with respect to the negative control, indicating that the extract did not affect the stages of cell division (Figure 5).

**Figure 6:**
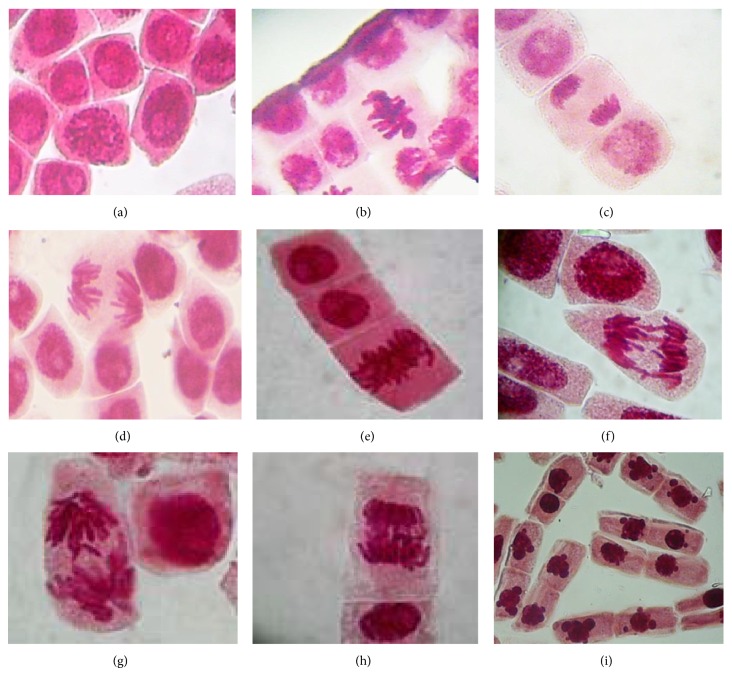
Microphotographs of the morphological alterations of* Allium cepa* cells treated with hot aqueous extract of* Achyrocline satureioides* (As-HAE). Normal cells in mineral water treatment (negative control): (a) cells in prophase, (b) cells in metaphase, (c) cells in telophase, and (d) cells in anaphase; (e) physiological aberrations: c-mitosis in paracetamol treatment (positive control); (f) cells with sticky chromosomes in As-HAE (4 mg/mL) treatment; (g) cells with delayed chromosomes in As-HAE (2 mg/mL) treatment; (h) cells with chromosomal bridges in As-HAE (4 mg/mL) treatment; (i) figures similar to apoptotic bodies in As-HAE (2 mg/mL) treatment.

Microscopic evaluation of cells showed physiological and clastogenic aberrations. The physiological aberrations observed were c-mitosis and sticky and delayed chromosomes, whereas the clastogenic aberrations were chromosomal bridges. Figures similar to apoptotic bodies were observed in interphase cells treated for 2 days with As-HAE (Figure 6). All microscopic alterations were found at a very low frequency. The highest rate was observed in the treatment with As-HAE at 4 mg/mL (2.20%); however there was no statistical difference with the negative control (1.00%).

Table 3 shows the micronucleus assay results obtained from Balb/c mice treated with As-HAE. No clinical signs of behavioral toxicity or mortality were observed in the animals treated with extract at every concentration. As expected, there was a significant increase in the frequency of MN in PCE from the positive control group treated with cyclophosphamide (*P* < 0.001). A dose-dependent increase in the frequency of MN in PCE from the treatments with As-HAE in comparison to the saline group was observed. The treatment with As-HAE at 200 and 500 mg/kg bw showed statistically significant difference with negative control (*P* < 0.005 and *P* < 0.001). In all treatments with As-HAE, a decrease in the PCE/NCE ratio with statistically significant difference compared to the saline group could be observed (*P* < 0.05). There were no sex-dependent changes in any treatment.

**Figure 7:**
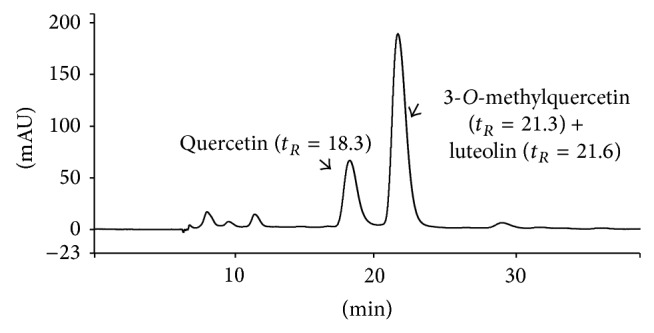
Qualitative HPLC analysis of enriched flavonoid fraction obtained from hot aqueous extract of* Achyrocline satureioides* (As-HAE).

The chemical evaluation of As-HAE-Ether was performed by means of a qualitative and quantitative HPLC-ESI-MS analysis, by following the previously used methodology by our research group for the assessment of the cold aqueous extract obtained from this same vegetal species [11]. This procedure allows identifying three flavonoids: quercetin (*t*_*R*_ = 18.3, parent ion [M − 1]^−^ = 301* m/z*), 3-*O*-methylquercetin (*t*_*R*_ = 21.3, [M − 1]^−^ = 315* m/z*), and luteolin (*t*_*R*_ = 21.6, [M − 1]^−^ = 285* m/z*) (Figure 7), which are the major flavonoids in polar extracts of this vegetal species [2, 3]. The amount of each compound identified in the extract is shown in Table 4 (quantification analysis). Data are expressed as mean ± standard deviation (SD) of three individual experiments. Thus, we established that luteolin is the highest proportion compound detected, followed by 3-*O*-methylquercetin and quercetin with the lowest recovery percentage.

## 4. Discussion

In this work, we extend the studies related to the toxic effects of hot aqueous extract (As-HAE) of* A. satureioides* carrying out both* in vitro *and* in vivo *assays.

In the* in vitro* assays, we demonstrated that As-HAE had toxic effects on human PBMCs. This extract results in more toxicity on these cells than cold aqueous extract affecting both the mitochondrial function and cell membrane integrity (as measured by MTT and trypan blue dye exclusion methods) (Table 1). Although As-HAE cause PBMCs damage which would lead to cell death, here we demonstrated by three apoptosis assays that extract did not induce Type 1 cell death (caspase-dependent). These results are in concomitance with data obtained for cold aqueous extract [11]. Perhaps the observed cell death induced by both extracts may be due to another type of programmed cell death [21]. This result is consistent with Barioni et al. [22], who investigated the leukocyte viability from circulating blood of rats following* in vivo* treatment with a hydroalcoholic extract of* A. satureioides* (whose major compounds identified were luteolin and quercetin) and showed that extract treatment did not cause apoptosis.

In the* in vivo* assays, we observed by the* Allium cepa* L. test that As-HAE exerted time and dose-dependent cytotoxic effects observed by roots length decrease and bulbs necrosis induction and MI decrease. Nevertheless, it is remarkable that As-HAE toxic effect was clearly reversed during mineral water exposure. The phase index analysis and the physiological and clastogenic aberrations evaluation would indicate that As-HAE did not induce genotoxic effects. These results are in concordance with a previous study performed with cold aqueous extract of* A. satureioides* [23]. Although* Allium cepa* L. test is a simple* in vivo* model, it has proven to be an efficient prescreening technique for later cytogenotoxic studies [24]. In order to complete the cytogenotoxic studies, we carried out the* in vivo* micronucleus test because it is a useful tool to evaluate genotoxicity of potential toxic substances and to detect chromosomal damage or mitotic disturbances in peripheral blood cells or bone marrow, providing valuable information. In contrast to that observed in* Allium cepa *L. test, the micronucleus assay revealed that As-HAE (200 and 500 mg/kg bw) exerted genotoxic effects on bone marrow of mice because of an increase in the micronucleated PCE frequency, at 24 h after injection. By contrast, the cold aqueous extract (up 250 mg/kg bw) did not induce genotoxic effects on bone marrow of mice [11]. Furthermore, in order to provide an assessment of the erythropoiesis rate and thus a cytotoxicity measurement, a percentage of PCE evaluation among total erythrocytes in bone marrow cells was included. The PCE/NCE ratio decreased in all treatments with As-HAE and showed alterations in hematopoiesis indicating cytotoxic effects on bone marrow cells, as observed for the cold aqueous extract of this species [11]. These cytotoxicity results are consistent with that found in* in vitro* assays and in* Allium cepa* L. test.

The chemical study of the As-HAE allowed identifying the flavonoids luteolin, quercetin, and 3-*O*-methylquercetin at higher concentrations than those found in cold aqueous extract (Table 4). We suggest that toxic effects induced by As-HAE both* in vitro* and* in vivo* could be due to a higher concentration of flavonoids with respect to cold extract. Polydoro et al. [3] found that the extracts of* A. satureioides *with highest concentration of flavonoids, especially quercetin, were more toxic on Sertoli cells.

Vargas et al. [25] demonstrated by the Ames test that hot aqueous extract of* A. satureioides* has* in vitro* genotoxic activity and that this effect was related to the presence of tannins and flavones, with certain hydroxylation patterns (5,7-hydroxyl substitution) such as quercetin, luteolin, and kaempferol. Moreover, Resende et al. [26] reported the genotoxic effects of luteolin and quercetin by the Ames test and showed that these compounds were mutagenic agents and caused base-pair substitutions, quercetin being more genotoxic than luteolin. Another* in vitro* study shows that quercetin induced chromosomal aberrations and sister chromatid exchanges in Chinese hamster ovary (CHO) cells [27]. Considering the above, quercetin and luteolin might be responsible of the cytogenotoxic effects induced by As-HAE.

In conclusion, we demonstrated that As-HAE induced cytotoxic and genotoxic effects being more toxic than cold aqueous extract. It is noteworthy that the amount of this vegetal species popularly consumed (around 1.5–2 mg/kg of body weight) is below the lowest concentration evaluated in the* in vivo* micronucleus test in the present work. However, given that the hot aqueous extract is the most used in folkloric medicine, its administration should be controlled because the misuse or abuse can cause host cell damage.

## Supplementary Material

The identification of flavonoids in an enriched fraction (ethyl ether) from the hot aqueous extract of Achyrocline satureioides (Lam.) DC (As-HAE) is shown below. By means of HPLC-ESI-MS/MS analysis we identified quercetin, luteolin and as 3-O-methylquercetin in this extract.
Fig. 1 shows the identification of quercetin (tR= 18.3) in the extract because its peak [M-1]= 301 was detected with its characteristic MS/MS rupture, which all are coincident with the data obtained for standard quercetin (Fig. 2). Fig. 3 shows the peak [M-1]= 285 corresponding to luteolin in the extract (tR= 21.6), which did not suffer rupture as the standard luteolin (Fig. 4). In Fig. 5 we can observe the peak [M-1]= 315 corresponding to 3-O-methylquercetin (tR= 21.3), which has a MS/MS rupture according to this structure since the loss of methyl was observed to give the peak 300 m/z (quercetin).

## Figures and Tables

**Table 1:** Cytotoxicity of hot aqueous extract of *Achyrocline satureioides* (As-HAE) on human PBMCs.

	TB CC_50_ (*µ*g/mL)	MTT CC_50_ (*µ*g/mL)
As-HAE	653	588
Cold aqueous extract^∗^	2660^∗^	>3000^∗^

TB: trypan blue dye exclusion method; ^∗^Sabini et al. [11].

**Table 2:** Macroscopic parameters analyzed in roots of *Allium cepa* L. after treatment with different concentrations of hot aqueous extract of *A. satureioides* (As-HAE) for 5 days and 2 days (with reversion).

Parameter	Treatments (*n* = 4 bulbs)													
	As-HAE (mg/mL) for 5 days							As-HAE (mg/mL) for 2 days (with reversion)						
	C (−)	C (+)	0.5	1	2	3	4	C (−)	C (+)	0.5	1	2	3	4
Mean roots number	47	37	46	55.5	31.5	38	51	40.5	34.5	49	47.5	45	38	46.5
Mean root length (mm) (±SEM)	25.35 (±2.435)	13.97 (±0.654)	6.18 (±0.381)	7.65 (±0.464)	9.02 (±0.790)	7.26 (±0.496)	6.79 (±0.567)	31.88 (±3.336)	17.55 (±0.936)	18.06 (±1.700)	13.17 (±1.279)	13.30 (±1.040)	13.29 (±1.213)	13.24 (±0.890)
Abnormalities														
H^a^	6	32	4	3	0	0	3	3	20	0	3	10	0	6
Ge^b^	0	0	0	4	0	5	13	0	0	1	9	6	6	20
Ne^c^	0	0	2	28	14	25	11	0	0	7	16	1	24	15
Tu^d^	2	5	0	0	0	0	0	4	5	1	0	3	0	0

^a^H: hook; ^b^Ge: gelling; ^c^Ne: necrosis; ^d^Tu: tumor.

**Table 3:** Genotoxic effects of hot aqueous extract of *A. satureioides* (As-HAE) on erythrocytes from bone marrow of mice.

Treatments	Animals^a^	PCE/NCE ± SD (TI)^b^ 24 h	MNPCE^c^(‰) 24 h
Negative control (saline solution)	6	1.38 ± 0.35	5.75 ± 1.77
Hot aqueous extract of *A. satureioides* (As-HAE) (mg/kg bw)			
100	6	0.23 ± 0.02^∗^	4.50 ± 1.54
200	6	0.22 ± 0.08^∗^	12.50 ± 1.31^∗^
500	6	0.30 ± 0.05^∗^	28.25 ± 2.35^∗∗^
Positive control (cyclophosphamide)	6	1.20 ± 0.60	26.50 ± 8.02^∗∗^

^a^Six mice were used per experimental group (3 males and 3 females). ^b^Toxicity index; ^c^micronucleated polychromatic erythrocytes. In all cases, 2000 polychromatic erythrocytes (PCE) per animal were analyzed; ^∗^ *P* < 0.01, ^∗∗^ *P* < 0.001 statistically significant difference from saline group (ANOVA Tukey test).

**Table 4:** Concentrations of quercetin, luteolin, and 3-*O*-methylquercetin present in AS-HAE-Ether.

	% P/P (mg compound/100 mg extract)		
	Luteolin	Quercetin	3-*O*-methylquercetin^#^
Hot aqueous extract	1.1894 ± 0.0012	0.1115 ± 0.0009	0.1785 ± 0.0023
Cold aqueous^∗^ extract	0.6028 ± 0.0002^∗^	0.0224 ± 0.0009^∗^	0.0405 ± 0.0003^∗^

^#^3-*O*-methylquercetin expressed in quercetin.

Data are expressed as mean ± standard deviation (SD) of three individual experiments. ^∗^Sabini et al. [11].

## Abbreviations

ESI: Electrospray ionization
FCS: Foetal calf serum
HPLC: High performance liquid chromatography
ME: Mercaptoethanol
MI: Mitotic index
MN: Micronuclei
MS: Mass spectrometry
MTT: 3,(4,5-dimethyl-2-thiazolyl)-2,5-diphenyl tetrazolium bromide
NCE: Normochromatic erythrocytes
PBMCs: Peripheral blood mononuclear cells
PCE: Polychromatic erythrocytes
TdT: DNA-terminal deoxynucleotidyl transferase
TUNEL: Terminal deoxynucleotidyl transferase biotin-dUTP nick end labeling.
