# Supplementary material for: In Vitro and In Vivo Cytogenotoxic Effects of Hot Aqueous Extract of Achyrocline satureioides (Lam.) DC
Source: Biomed Res Int. 2015 May 11;2015:270973. doi: 10.1155/2015/270973 (PMC4442415; doi:10.1155/2015/270973)
Supplement: Supplementary file 1 — The identification of flavonoids in an enriched fraction (ethyl ether) from the hot aqueous extract of Achyrocline satureioides (Lam.) DC (As-HAE) is shown below. By means of HPLC-ESI-MS/MS analysis we identified quercetin, luteolin and as 3-O-methylquercetin in this extract. Fig. 1 shows the identification of quercetin (tR= 18.3) in the extract because its peak [M-1]= 301 was detected with its characteristic MS/MS rupture, which all are coincident with the data obtained for standard quercetin (Fig. 2). Fig. 3 shows the peak [M-1]= 285 corresponding to luteolin in the extract (tR= 21.6), which did not suffer rupture as the standard luteolin (Fig. 4). In Fig. 5 we can observe the peak [M-1]= 315 corresponding to 3-O-methylquercetin (tR= 21.3), which has a MS/MS rupture according to this structure since the loss of methyl was observed to give the peak 300 m/z (quercetin). [file 270973.f1.doc]

**Annex**

**Figure 1:** MS and MS/MS of ion 301 identified as quercetin in the extract

quercetin

**Figure 2**: MS and MS/MS of standard quercetin

quercetin

**Figure 3**: MS and MS/MS of ion 285 identified as luteolin in the extract

MS ión 285 luteolin

MS/MS ión 285

**Figure 4:** MS and MS/MS of standard luteolin

luteolin

luteolin

**Figure 5:** MS and MS/MS of ion 315 identified as 3-*O*-methylquercetin in the extract

MS ión 315 3-*O*-methylquercetin

MS/MS ión 315
